# Botulinum toxin in fracture management: a scoping review

**DOI:** 10.1007/s00590-025-04295-4

**Published:** 2025-06-04

**Authors:** Emmanuel Spanoudakis, Themistoklis Vampertzis, Farokh Wadia, Darius Rares Rad, Christina Barmpagianni, Ioannis A Zervos, Eleftherios Tsiridis, Nikiforos Galanis

**Affiliations:** 1 University Hospital Southampton NHS Foundation Trust, Southampton, United Kingdom; 2 Aristotle University of Thessaloniki, Thessaloniki, Greece; 3 Surrey and Borders Partnership NHS Foundation Trust, Leatherhead, United Kingdom; 4 Papageorgiou General Hospital, Thessaloniki, Greece; 5 Aristotle University of Thessaloniki, Thessaloniki, Greece

**Keywords:** Botulinum toxin, Fracture healing, Muscle paralysis, Maxillofacial fractures, Orthopaedic trauma, Fracture immobilisation

## Abstract

**Purpose:**

Using botulinum toxin (BoNT) as an adjunct in fracture management is a novel approach with the potential to improve clinical outcomes, particularly in atypical fractures and patients with concurrent neuromuscular disorders. This scoping review explores the literature on BoNT’s effectiveness in facilitating fracture healing and immobilisation across various fracture types.

**Methods:**

The PubMed, Embase, and Cochrane databases were searched with defined operators. Two investigators conducted independent searches, which were combined. Animal studies, case reports, case series, cohort reviews and randomised control trials were included.

**Results:**

Fifty studies were identified for screening, from which 15 studies were included in the review. The findings highlight mixed outcomes in BoNT’s role in enhancing bone healing and reducing muscle-induced displacement. While BoNT injections demonstrated potential benefits in specific cases, such as atypical fractures, periprosthetic fractures and fractures in patients with motor dysfunctions, results from animal studies were inconsistent, showing varied effects on callous formation and bone mineral density.

**Conclusion:**

Clinical applications of BoNT in fracture management indicate its utility as an immobilisation agent to promote fracture healing and reduce complications. However, this review highlights that further research is necessary to bridge the gap between experimental and clinical studies and to clarify BoNT’s effectiveness in different use cases.

**Supplementary Information:**

The online version contains supplementary material available at 10.1007/s00590-025-04295-4.

## Introduction

Botulinum toxin (BoNT) has emerged as a promising adjunct therapy in managing orthopaedic and maxillofacial fractures in conjunction with traditional modalities such as immobilisation, operative fixation and rehabilitation. It has shown potential for improving outcomes in patients with atypical fractures and those with neuromuscular disorders. However, animal studies have demonstrated both beneficial and detrimental effects on bone healing. This highlights the need for further clinical research to establish treatment recommendations for using BoNT and to bridge the gap between animal and clinical studies. As such, this scoping review aims to evaluate the evidence surrounding using BoNT in fracture management. 

## Methodology

### Search strategy

A comprehensive search of publications was conducted using the EMBASE and Medline PubMed databases as per the Preferred Reporting Items for Systematic Reviews and Meta-Analyses for Scoping Review (PRISMA-ScR) guidelines [1, 2]. Existing reviews (systematic or scoping) were evaluated in the MEDLINE PubMed, EMBASE, Cochrane Library (CENTRAL) and databases. The following keywords and MeSH terms were included in the search strategy: “Botulinum Toxins”, “Botulinum Toxin”, “Botulinum Toxins, Type A”, “Botulinum Toxin, Type A”, “Fracture Healing”, “Fractures, Bone”, and “Fracture, Bone”. MeSH terms were derived from PubMed and were subsequently modified to expand the search. We used Boolean operators (OR, AND) to maximise the sensitivity of the search. A complete breakdown of the search strategy can be found in online Appendix 1. 

### Inclusion/exclusion criteria

Inclusion criteria comprised original publications with a PubMed ID evaluating the use of botulinum toxin in fracture management; studies had to explicitly state in the abstract/title the use of botulinum toxin in fracture healing. This included orthopaedic and maxillofacial fractures. We also included experimental animal studies to expand our search. We excluded abstracts and letters to the editor.

### Results

After the initial literature search, 50 studies were identified for screening. After a review of abstracts, 14 full-text articles were assessed for inclusion and exclusion criteria. Of the 14 articles sought for retrieval, one was not retrieved, and two were excluded as they did not relate to using BoNT in fracture management. Four additional studies were found on further review of citations and other literature, all meeting the inclusion criteria. The final 15 studies included one randomised control trial, one retrospective cohort review, three case series, five case reports, and five animal studies. A complete summary of the articles included can be found in Table 1.

**Table 1:** Summary of reviewed articles including outcomes

Author	Year	Country	Study type (Level of Evidence)	Subjects	Fracture location	Internal fixation?	Botulinum injection site	Botulinum toxin dose	Control	Summary of outcomes
Akbay et al.	2014	Turkey	Case report (V)	One male	Mandible (Condylar neck)	N	Masseter, temporalis, and pterygoideus medialis muscles	32 IU *(6 IU, 20 IU, 6 IU respectively)*	n/a	Fusion of mandibular condyle was achieved after one month
Aydin et al.	2012	Turkey	Animal study	18 rats	Femoral shaft (Bilateral)	Y	Right Anterior & Posterior Muscular Compartments	8 IU *(4 IU & 4 IU respectively)*	No injection	Increased biomechanical and histologic healing with decreased callous diameter was found on the BoNT-injected side 28 days after administration
Canter et al.	2007	Turkey	Case series (IV)	Six males and four females	Mandible (Condylar neck)	N	Masseter, anterior fibres of the temporalis, medial and lateral pterygoid muscles	100 IU *(30 IU, 30 IU, 40 IU respectively)*	n/a	Successful healing, with no loss of reduction. Return of muscular contractions after 3–4 months
Chee et al.	2004	UK	Case report (V)	One female	Robert Jones fracture	N	Gastrocnemius & Tibialis Posterior	Not specified	n/a	Botulinum toxin injections failed to promote bone healing
Chen et al.	2010	Australia	Case report (V)	One male	Clavicle	Y	Pectoralis Major; Anterior Deltoid; Trapezius; Levator Scapulae	180 IU *(60 IU, 40 IU, 50 IU, 30 IU, respectively)*	n/a	Cessation of motor tics for 3–4 months with successful healing of the fracture
Ellegaard et al.	2013	Denmark	Animal study	8 Female rats	Tibia (Closed)	Y	Ipsilateral hamstring, quadriceps & calf muscles	6 IU Total*	n/a	Reduced callous formation, bone mineral content & bone mineral density in unloaded (BoNT) group
Hamdy et al.	2016	Canada	RCT (I)	125 Children	Femur or Tibia*	N *(external fixation)*	Quadriceps & Hamstrings OR Gastrocnemius & Soleus	10 IU/kg	0.9% NaCl	Lower complication rate in BoNT group; no significant differences in adverse events or bone healing rates between groups
Hao et al.	2012	China	Animal study	44 Rats	Femoral Osteotomy (Bilateral)	Y	Unilateral Quadriceps	2 IU	0.9% NaCl	No mature osseous callouses or woven bone on the BoNT-treated side after eight weeks
Konas et al.	2011	Turkey	Case series (IV)	Eight males and two females	Complex or Communited Maxillofacial Fractures	Y	Masseter, temporalis, and medial and lateral pterygoid muscles	100 IU	n/a	Complete bone healing in all fractures at (avg.) 12.7-month follow-up; no re-operations
Lapow et al.	2023	USA	Case report (V)	One female	Humeral Shaft Fracture (Closed; Distal Third)	Y	Right Triceps Brachii, Biceps Brachii and Brachialis Muscles	200 IU *(100 IU, 75 IU, 25 IU, respectively)*	n/a	Formation of bridging callous and bone formation, as well as return of motion at 3 months following BoNT injection
Shilt et al.	2021	USA	Retrospective case series (IV)	Five males	Proximal Hamstring Avulsion	Y *(all except one patient)*	Hamstring muscles	100 IU *(except for conservatively managed patient—200 IU)*	n/a	Radiograph-confirmed healing of avulsion fractures in all patients with a return to previous activity at a mean of 32 weeks
Shin et al.	2018	South Korea	Retrospective Cohort Review (III)	Six males and two females	Bilateral sagittal split ramus osteotomy	Y	Bilateral masseter muscles	50 IU *(25 IU per side)*	No injection	Lower rate of mandibular plate fracture in BoNT injections vs. no injections
Tukel et al.	2020	Turkey	Animal study	48 Male rabbits	Mandible (Unilateral)	Y	Masseter Muscles	10 IU	0.9% NaCl	Improved fracture healing after 21 days compared to saline-injected controls
Vampertzis et al.	2024	Greece	Animal study	28 Rats	Clavicle (Unilateral)	N	Ipsilateral Sternocleidomastoid Muscle	4 IU	0.9% NaCl	No difference in clavicle fracture healing between BoNT and controls
White et al.	2022	USA	Case report (V)	One female	Periprosthetic scapular spine fracture	Y	Deltoid muscle	8 IU	n/a	Fracture union at three months with excellent return to function at one year

^*^Divided between muscles at different time frames surrounding fracture; 2.5 IU (0.5 IU, 1 IU, 1 IU) 5 days pre-fracture; 2 IU (0.5 IU, 0.5 IU, 1 IU) 2 weeks post-fracture; 1.5 IU (0.5 IU, 0.5 IU, 0.5 IU) 6 weeks post-fracture

^**^Distraction osteogenesis via osteotomy & external fixator

## Discussion

### Animal studies

Multiple animal studies (involving adult male rats) investigated the effect of temporary muscle paralysis on bone healing following fractures, with equivocal results. The mixed results observed in animal studies examining the impact of botulinum neurotoxin (BoNT) on bone healing post-fracture can be partly attributed to variations in anatomical targeting and the extent of muscle paralysis [3, 4].

Hao et al. investigated the effect of BoNT-induced local muscular atrophy and dysfunction on surgically induced mid-femoral shaft fractures in male rats, with contralateral controls consisting of BoNT injections vs. 0.9% saline injections, respectively. Femoral fixation was performed with intramedullary nailing. Serial X-rays were taken to monitor bone healing progress and periosteal callous formation, as well as three-point bend testing and histopathological evaluation. Overall, the authors demonstrated that localised BoNT-induced quadriceps atrophy led to impaired periosteal callous formation, incomplete gap reduction, and histologically immature bone regeneration in male rats with femoral shaft fractures, highlighting some possible adverse effects of muscular unloading on fracture healing.

Aydin et al. investigated the administration of BoNT in rats with surgically induced closed femoral shaft fractures. BoNT was injected into the right thigh’s anterior and posterior muscle compartments in all rats; the left thighs served as the control. Callous size, three-point bending test and subsequent histopathological evaluation of fracture healing (as per the Lane and Sandhu Scoring System) were assessed following the rats’ death [5]. The authors reported improved bone healing following BoNT injection into both anterior and posterior thigh compartments, with enhanced trabecular organisation and increased elastic modulus, which they attributed to BoNT’s potential vasodilatory and angiogenic effects that may enhance fracture site perfusion [6]. The study notes that its findings contradict existing literature investigating the effects of muscle paralysis (via sciatic nerve resection) on post-traumatic bone loss, with Aydin et al. demonstrating improved fracture healing [7].

Vampertzis et al. described using BoNT in the sternocleidomastoid muscle in rats with conservatively managed right-sided clavicle fractures. Radiographic and histopathological assessments of bone and sternocleidomastoid muscle found no significant difference in fracture healing between the control and BoNT groups. However, the authors note increased cell necrosis and atrophy in the sternocleidomastoid muscles adjacent to the fracture site. This raises concerns regarding long-term muscle integrity, given that potentially irreversible adverse effects in muscle have been noted in humans, such as neurogenic atrophy and neuromuscular junction degeneration [8, 9].

Ellegaard et al. investigated the effect of parathyroid hormone (PTH) on bone healing in different loading conditions. They used ovariectomised rats who had undergone tibial shaft fractures and subsequent internal fixation, simulating different loading conditions by injecting BoNT into the ipsilateral hamstrings, quadriceps and calf muscles. The unloaded group (BoNT) demonstrated reduced bone mineral content (BMC) and density (BMD) at all time points following fracture, and no interaction was detected with PTH administration.

The anatomical specificity of BoNT injections, as outlined by Yi et al. in their mapping of motor endplates in the latissimus dorsi and sartorius muscles, is particularly relevant in this context. The authors emphasise the importance of precision in BoNT injection location for specific muscle modulation without compromising the biomechanical environment necessary for optimal bone healing [3, 4]. As such, integrating this anatomical knowledge may bridge the gap between experimental animal studies and future clinical practice.

Finally, Tukel et al. studied 48 male rabbits to assess the effects of BoNT injection on bone healing in a mandibular fracture model with plate fixation. They measured radiological, histopathological and biomechanical outcomes. The control group (0.9% NaCl injections) showed significantly lower mean BMD, failure load and bending modulus than the BoNT group, indicating better fracture healing and biomechanical properties in the latter. The BoNT group also had superior histopathological results compared to the control group. The authors acknowledge that their findings contradict existing animal studies of orthopaedic fractures [11, 21]. Superior bone healing in the BoNT group was attributed to the reduced masticatory bone strain on the mandible, given the high displacing forces of the masseter on bone segments, particularly without large load-bearing plates. The authors also concede that while using BoNT may be clinically valuable in cases of limited bone contact between fractured segments or pathological increases in the forces of mastication, further (human) studies are required to determine the concrete use cases.

Overall, the mixed outcomes from animal studies suggest that BoNT’s impact on bone healing can vary; this could be explained by inadequate precision of injection location, as highlighted by Yi et al. in their mapping of neural arborisation and motor endplate zones [3, 4]. This highlights the need for standardised and anatomically informed application protocols before BoNT can be included in orthopaedic or maxillofacial guidelines.

### Use of botulinum toxin as immobilisation in concurrent motor dysfunction

We found three case reports on BoNT for patients with neuromuscular disorders (NMD) and acute long-bone fractures. Given the unpredictability of mechanical loading in NMD, it is important to determine if BoNT adequately reduces displacing forces to improve fracture healing and to determine possible post-operative complications.

Lapow et al. report a right distal humerus fracture in a patient with Parkinson’s disease (PD) and psychosis, who underwent open reduction and internal fixation (ORIF) with a Gerwin modification. Fifteen days post-injury, she faced wound breakdown and an open periprosthetic fracture from thrashing episodes linked to her PD psychosis. She underwent washout, removal of metalwork, and ORIF of a T-condylar humerus fracture via a paratricipital approach with ulnar nerve neuroplasty. Post-operatively, she received BoNT injections into the triceps, biceps and brachialis for better elbow immobilisation and PD management. Anatomical studies could guide BoNT injections for optimal motor endplate zones, helping reduce muscle force displacements, as evidenced by Yi et al. in muscle spasticity [4]. Three months later, there was no wound breakdown, dehiscence, or infection; X-rays showed callous formation and bone bridging at fracture sites, with no loose metalwork. The median, ulnar and radial nerves maintained good motor and sensory function. However, follow-up was not possible due to the patient’s unrelated death.

Chen et al. report a paediatric case of a left-sided clavicle fracture and a concurrent tic disorder worsened by peri-operative pain. Initial conservative management showed non-union on follow-up X-rays. The patient underwent ORIF, which led to a haematoma and wound dehiscence on Day Two post-op. A second operation was performed for haematoma evacuation and re-suturing. In collaboration with pain specialists and neurologists, BoNT injections into the ipsilateral pectoralis major, deltoid, trapezius, and levator scapulae were administered as the wound issues were deemed to be caused partly by the tic disorder. His tic activity decreased significantly, and the fracture united.

Chee et al. report a paediatric case of a Robert Jones fracture in a cerebral palsy patient with ipsilateral monoplegia and equinovarus foot deformity. The fracture followed a fall and was treated conservatively with a below-knee weight-bearing cast for 4 months, but an atraumatic re-fracture occurred two months later. The equinovarus deformity was corrected under anaesthesia to alleviate pressure on the foot. Nine months post-injury, BoNT injections in the gastrocnemius and tibialis posterior muscles failed to promote healing, necessitating the subsequent Baumann procedure with tibialis posterior tendon division. As such, the patient was successfully treated for a fifth metatarsal stress fracture through surgical deformity correction, with unsuccessful reduction of deforming forces from BoNT administration [10].

These cases highlight BoNT’s potential as an adjunctive tool in complex scenarios where conventional immobilisation is inadequate. Therefore, BoNT could be integrated into emerging clinical pathways for patients with movement disorders or spasticity as an adjunct to traditional immobilisation methods for reducing deforming forces in the pre- and post-operative periods. However, its incorporation into current clinical guidelines presents challenges, such as clear patient selection criteria, timing, and muscle targeting criteria, ideally informed by anatomical studies and clinical trials.

### Use in atypical fractures

Hamdy et al. conducted a randomised trial on the actions of BoNT injections in 125 paediatric patients undergoing unilateral limb-lengthening distraction osteotomies (DO) on the tibia or femur. We adopt the AO definition of a fracture as any disruption in the bone cortex, acknowledging that an osteotomy is not a traditional fracture. BoNT injections were compared to a placebo (0.9% NaCl). They were administered in the anterior and posterior muscles for femoral osteotomies and in the gastric–soleus complex for tibial osteotomies. The primary outcome measures included medication use, subjective pain and quality of life scores, physical function markers (weight-bearing status, ambulation and range of motion), complication rates and bone healing index (BHI) scores.

On day 1 post-op, maximum and mean pain scores were lower in the BoNT group, showing statistical significance; no pain ≥ 3/10 (VAS) was reported after day four. The authors attempted to stratify pain scores by distraction level but noted insufficient sample size. They also found lower analgesic and anti-spasmodic use in the BoNT group vs control in the immediate post-operative period, though not statistically significant. Quality of life scores showed no significant differences at any time points between BoNT and control. Still, there were equivocal clinical differences between parent and child-reported quality of life on post-op days two, three, and four.

All adverse events reported were expected complications of the external fixation device, with around half being pin-site infections. The authors noted no serious adverse events from BoNT usage, which had a 10% lower incidence of adverse events than the control group. When stratified by location, the BoNT group showed significantly lower rates of pin-site infections in tibial osteotomies compared to the control group. However, this was not seen in femoral osteotomies. BHI scores were similar in both groups.

No significant differences in ambulation or weight-bearing were found between BoNT and control at any time. Similarly, no ankle or knee range of motion or function differences were observed at 3 or 6 months. Both groups showed initial lag at 3 months, which improved by 6 months. This is positive, given the negligible clinical difference in muscle function, despite concerns from animal studies about the irreversible impact of BoNT on muscle mass and composition [8, 9, 11, 12].

Shilt et al. studied five male patients (ages 12–17; mean age 15) who underwent surgery after unsuccessful conservative treatment for hamstring avulsion injuries at the ischium. They used BoNT injections for controlled immobilisation, which proved more effective than complete immobilisation. Four patients received ORIF (avulsions displaced > 1.4 cm) and 100 IU BoNT, while one with less displacement received a bone marrow aspirate and 200 IU BoNT. Only two patients used knee braces locked at 20° flexion; the first two braced for one and three weeks, shorter than the BoNT treatment duration. Follow-ups included imaging, rehabilitation progress and return-to-sport times.

In this context, BoNT injection is supported by evidence that external bracing often causes falls postoperatively and complicates recovery [13–15]. While it is not a standard post-operative therapy, BoNT can serve as adjunct for immobilisation in neuromuscular conditions; as such, it has demonstrated utility in protecting tendon repairs in the hand, the Achilles tendon, and the distal biceps tendon [15–17]. Shilt et al. compare their return-to-sport times with the literature, noting that their method’s minimal post-operative bracing is advantageous when used with BoNT.

The rationale for BoNT in atypical fractures—particularly in reducing complications from external bracing and uncontrolled muscle forces—addresses current challenges in clinical management. While BoNT is absent in current immobilisation guidelines, its role as an immobilisation agent in specific cases could enhance protocols in more complex patients, such as those with neuromuscular disorders. Additional research may guide its role in atypical fractures where current immobilisation techniques are inadequate.

White et al. published a case report on BoNT injections as an adjunct to the surgical management of a periprosthetic scapular spine fracture. The patient was a 73 year-old female who sustained a Levy type two scapular spine fracture after sustaining a fall 6 months following primary reverse shoulder arthroplasty (rTSA; for end-stage glenohumeral osteoarthritis). She had no neurovascular compromise following her injury and was able to use all three heads of her deltoid muscle (limited by pain). She received an 8 IU BoNT injection on day three post-ORIF to reduce shear forces across the fracture site; this was in addition to shoulder immobilisation. X-ray evidence of healing was seen at six weeks, two months, and seventeen months following ORIF; deltoid power was reported to be 4/5 at the two-month follow-up, so this is when her active range of motion physiotherapy commenced. She was noted to have a union of her fracture and 5/5 deltoid function at 17 months.

The authors note that periprosthetic spine fractures are common following rTSA and often require ORIF to manage and have difficulty healing [18, 19]. Given that a cause of increased scapular spine fracture risk in rTSA patients is the increased shear forces by the deltoid muscle over the rigid fracture construct due to the shifting of the glenohumeral centre of rotation medially and distally, BoNT injection in the deltoid muscle would reduce these forces in the context of a spine fracture. The success of this targeted approach aligns with anatomical studies that map intramuscular neural arborisation, such as Yi et al. (2022), who demonstrated the clinical relevance of understanding nerve distribution in the latissimus dorsi to improve BoNT efficacy in flap reconstruction [3]. Similar anatomical mapping can aid in optimising BoNT delivery in orthopaedic applications, which would be a valuable tool given the high risk of non-union and poorer patient outcomes in periprosthetic scapula spine fractures and other similar clinical contexts.

### Use in maxillofacial fractures

Canter et al. studied 100 IU BoNT injections in the masseter, anterior temporalis and pterygoid muscles for treating ipsilateral neck fractures of the mandible alongside an asymmetric splint. The injections induced muscle paralysis in all ten patients (six male, four female), with no adverse outcomes reported. The mandibular rami and condylar processes were well-aligned, and no significant dislocation of the condylar head occurred. The authors found splint application simpler than expected in these fractures, referencing the effectiveness of the BoNT injection. They also suggest that BoNT can decrease the contracting muscle’s displacing forces on the fractured condylar segment, facilitating splint application and reducing additional fracture displacement.

Konas et al. reviewed splint-assisted reduction in complex maxillofacial fractures involving ten patients with pre-operative malocclusion. They underwent intermaxillary fixation after reduction under general anaesthesia. Three of these patients, with bilateral or displaced condylar neck fractures, received BoNT injections in mastication muscles following Canter et al.’s protocol. At the 12-month follow-up, no significant malocclusion was noted, though one patient showed signs of condylar resorption at 13 months due to avulsion-related soft tissue damage. The study does not compare BoNT usage but confirms its safety in treating involved mandibular condyle fractures, adding to Canter et al.’s findings [20].

Shin et al. conducted a retrospective cohort study on 16 patients using BoNT to reduce post-operative plate fractures after bilateral sagittal split ramus osteotomy for mandibular advancement. They found a significantly lower rate of plate fractures in the BoNT group compared to the control and a smaller, non-significant change in the SNB angle at six months postoperatively. The authors suggest this is due to reduced masseter muscle power, which lowers stress on the mandibular condyle.

Akbay et al. reported a case of a 3 year-old boy with an incomplete fracture of the mandibular symphysis and a displaced condylar fracture. Due to his injuries, he did not receive ORIF but was treated with an occlusal splint (as in Canter et al.). Given the ongoing condylar angulation, he had a BoNT injection in the ipsilateral masseter, temporalis and pterygoideus medialis. The authors concluded that the reduced masticatory strength from BoNT injection helped reduce the condyle in this conservatively managed fracture.

The consistency of results across the above studies highlights BoNT’s clinical utility in maxillofacial trauma, particularly in mandibular condyle fractures where muscle forces interfere with initial reduction. Given the findings in the existing literature, BoNT could be recommended in future guidelines as a viable adjunct in specific cases of maxillofacial fractures—especially where mechanical reduction alone is inadequate. However, updating existing clinical practices will require further research, with an emphasis on anatomical precision in BoNT injection sites [3, 4].

## Conclusion

BoNT in fracture management has demonstrated benefits and limitations depending on the circumstances of fracture and additional neuromuscular conditions. BoNT has shown a positive impact on maxillofacial trauma, not least in part due to its ability to suppress the muscles of mastication, leading to reduced displacing forces on mandibular condyle fractures [20, 22–24] and aiding in fracture reduction. While this effect has demonstrated promise in atypical orthopaedic fractures, such as those involving disorders of motor dysfunction [13, 14] and atypical fractures [25, 26], animal studies that simulate more typical long-bone trauma have shown more mixed results, [11, 12, 21]. Furthermore, the integration of anatomical insights, such as the intramuscular neural arborisation of the latissimus dorsi and the precise localisation of motor endplates in the sartorius muscle, reinforces the importance of precision in BoNT application [3, 4]. Overall, the limited and mixed evidence suggests a need for caution in treatment recommendations. As such, the application for further clinical trials before broad treatment guidelines is established.

## Supplementary Information

Below is the link to the electronic supplementary material.

Supplementary file1 (DOCX 14 kb)

## Data Availability

No datasets were generated or analysed during the current study.
