# Supplementary material for: Botulinum toxin in fracture management: a scoping review
Source: Eur J Orthop Surg Traumatol. 2025 Jun 4;35(1):233. doi: 10.1007/s00590-025-04295-4 (PMC12137460; doi:10.1007/s00590-025-04295-4)
Supplement: Supplementary file 1 — Supplementary file1 (DOCX 14 kb) [file 590_2025_4295_MOESM1_ESM.docx]

**Appendix 1**

Searching operator for systematic review in CENTRAL:

| Search Terms | Results |
| --- | --- |
| Botulinum Toxins" OR "Botulinum Toxin" OR "Botulinum Toxins, Type A" OR "Botulinum Toxin, Type A":ti,ab,kw AND "Fracture Healing" OR "Fractures, Bone" OR "Fracture, Bone":ti,ab,kw (Word variations have been searched) | 0 |

Searching operators in Ovid (Searching MEDLINE and EMBASE Databases):

| Database: Embase <1974 to 2024 November 01>, Ovid MEDLINE(R) ALL <1946 to November 01, 2024> | |
| --- | --- |
| Search Terms/Action | Results |
| (("Botulinum Toxins" or "Botulinum Toxins, Type A") and ("Fracture Healing" or  "Fractures, Bone" or "Fracture, Bone")).mp. [mp=ti, ab, hw, tn, ot, dm, mf, dv, kf, fx,  dq, bt, nm, ox, px, rx, an, ap, ui, sy, ux, mx] | 13 |
| (("Botulinum Toxin" or "Botulinum Toxin, Type A" or "Botulinum Toxins" or  "Botulinum Toxins, Type A") and ("Fracture Healing" or "Fractures, Bone" or  "Fracture, Bone")).mp. [mp=ti, ab, hw, tn, ot, dm, mf, dv, kf, fx, dq, bt, nm, ox, px, rx,  an, ap, ui, sy, ux, mx] | 66 |
| remove duplicates from 2 | 50 |
